# Identifying vaccination rates of adult patients in ambulatory care clinics

**DOI:** 10.1177/2050312120935461

**Published:** 2020-06-20

**Authors:** Amber Y Darr, Sarah Gottfried

**Affiliations:** 1 Department of Pharmacy Practice, Bernard J. Dunn School of Pharmacy, Shenandoah University, Winchester, USA; 2 Walreens, Purcellville, USA

**Keywords:** Vaccination, immunization, ambulatory care, herpes zoster, hepatitis B, tetanus, pneumococcal

## Abstract

**Background:**

While pharmacists have provided vaccinations to patients in the community pharmacy setting, pharmacist involvement within the medical office setting is not well documented in the literature. The American Society of Health-System Pharmacists reports that ambulatory care pharmacists are screening for and administering vaccinations at a declining rate, despite standards of practice. Vaccination rates for adults 19–64 years of age remain low, based on Healthy People 2020 goals, putting them at risk for vaccine-preventable diseases.

**Objectives:**

The aim of the study was to assess vaccination rates of ambulatory care pharmacy clinic patients aged 19–64 years and to compare the rates between three clinics and to Healthy People 2020 goals.

**Methods:**

This was a baseline retrospective analysis of vaccination rates for patients aged 19–64 years who attended at least one pharmacy clinic visit at one of the three medical office practices. Age, sex, medical conditions, cigarette or alcohol use, immunosuppressive medications, and vaccines recommended and received were recorded. Vaccination status was assessed according to the Advisory Committee for Immunization Practices recommendations. Data were collected from January 2016 to March 2017. The percentage of eligible patients who received each vaccine was determined overall and for each clinic.

**Results:**

There were 240 patients who met the inclusion criteria, with a mean age of 52.8 years. The percentage of patients with vaccination documented in the medical record was 25% for pneumococcal conjugate, 35.7% for pneumococcal polysaccharide, 26.9% for zoster vaccine live, 6.4% for hepatitis B, and 50.6% for tetanus toxoid, reduced diphtheria toxoid, and acellular pertussis. Vaccination rates for pneumococcal conjugate, pneumococcal polysaccharide, and zoster vaccine live were below established Healthy People 2020 goals.

**Conclusion:**

Vaccination rates remain low in adults 19–64 years of age. Ambulatory care pharmacists should consider assessing vaccination status during clinic visits as a component of comprehensive vaccination programs.

## Background

Pharmacists have provided vaccinations within the community pharmacy setting for over 20 years.^1^ Vaccination rates have increased due to pharmacist involvement with advocacy, education, and administration of vaccines within the adult population.^1,2^ While adoption of immunization protocols and procedures have been implemented as a standard of practice in community pharmacies across the United States, the activities of pharmacists vaccinating within the ambulatory care setting are not well documented.^1,2^ A systematic review and meta-analysis found that pharmacist involvement in vaccination education, facilitation, and administration resulted in an increase in vaccine coverage when compared to vaccine provision by other providers without pharmacist involvement.^3^ The American Society of Health System-Pharmacists (ASHP) 2001 survey of the responsibilities of ambulatory care pharmacists in managed care and integrated health systems reported pharmacist activities in screening for vaccinations declined by 24% and activities in administering vaccinations declined by 27%.^4^ A 2004 survey further reported the percentage of ambulatory care pharmacists who provide screening for vaccinations to be 28% and administration of vaccinations to be 14%.^5^ Pharmacists within Veterans’ Administration facilities reported no activity with immunization screening.^5^

Vaccination coverage in the United States for pneumococcal disease among adults 65 years of age or above was reported to be approximately 67% in 2016 and below the 90% Healthy People 2020 goal for that age group.^2,6^ The overall rate of vaccination for patients between the ages of 19 and 64 years remained at 24% and below the established Healthy People 2020 goal of 60% for this age group.^2,7^ In 2015, herpes zoster vaccination rates among adults 60 years of age or above met the Healthy People 2020 goal of 30%,^4^ and a rate of approximately 24% among patients 60–64 years of age in 2016.^6,8^ The Affordable Care Act (ACA) and the implementation of immunization protocols as a standard of practice in community pharmacies have contributed to increased patient access.^9^ However, the ACA has led to physician dissatisfaction with reimbursement through Medicaid and Medicare, and lack of knowledge of vaccine-specific provisions and uncertainty of insurance coverage has contributed to physicians not recommending adult vaccinations.^10^ The National Vaccine Advisory Committee (NVAC) adopted updated standards for adult immunization, which states that all health care providers, regardless of practice setting, should assess immunization status as a standard of care for all adult patients.^11^

Barriers to increasing vaccination rates of adult vaccines have been identified to include financial issues, limited access, and patients’ lack of knowledge regarding eligibility or need for a vaccine.^9^ Hurley and colleagues^10,12^ determined that patients are either refusing vaccine recommendations or providers are not recommending vaccines due to financial reasons, including lack of insurance coverage or low reimbursement. A 2017 survey of outpatient pharmacists (identified as directly dispensing pharmaceuticals to adults) and clinicians (physicians, physician assistants, and nurse practitioners) reported differences in barriers that prevent routine assessment and administration of vaccinations for adult patients > 19 years of age, concluding that discrepancies in actual practices of providers persist.^13^ Barriers to routine vaccine assessment that were most identified by clinicians and pharmacists were similar but ranked differently by level of importance. Clinicians acknowledged that scope of practice (68.9%), inadequate expertise (41.4%), lack of time or staff support (37.1%), low reimbursement (30.7%), and vaccination being a low priority (23.1%) as the most common barriers to routine vaccine assessment.^13^ Whereas, outpatient pharmacists reported lack of time or staff support (69.4%), inadequate reimbursement (54.8%), scope of practice (34.5%), low priority (24.4%), and inadequate expertise (24.4%) as the most important barriers.^13^ Interestingly, clinicians felt routine vaccination assessment is outside of their scope of practice and lack of expertise as more of a barrier than pharmacists. Regarding barriers to vaccine administration, clinicians and pharmacists also differed in their opinions of what was most important. Clinicians identified lack of storage and handling equipment (54.4%), outside the scope of practice (53%), and inadequate staffing or time (48.1%) as the top three barriers to routinely administering vaccines.^13^ In contrast, outpatient pharmacists reported that inadequate staffing or time (44.8%), inadequate reimbursement (28.8%), and lack of storage (25.9%) as being most common.^13^ While both groups share the concern of inadequate time or staffing to facilitate the administration of vaccinations, pharmacists in this study did not feel that the administration of vaccines was outside their scope of practice. Of those pharmacists and clinicians who administer vaccines, < 40% submit records to the state immunization information system (IIS).^13^

ASHP identifies the promotion and active administration of vaccines in all practice settings as one important role of the pharmacist.^14^ Primary care physicians and general internists have reported assessing the vaccination status of their patients at every visit less than 33% of the time and tend to refer vaccinations to pharmacies or the health department.^12^ One retrospective chart review of a large group practice reported increased influenza vaccination rates after the implementation of a pharmacist-run immunization program in cardiovascular patients.^15^ Expanding pharmacist patient care services to include vaccine promotion and administration within ambulatory care clinics is an example of the expanding role of pharmacists and contribution to primary care practice.^14^ Despite clear recommendations from Advisory Committee for Immunization Practices (ACIP) and guidelines set by ASHP and NVAC, there is a lack of outcomes research documenting the impact of pharmacists providing vaccination services in ambulatory care clinics.^1,2,7,8^ The success of community pharmacists providing vaccinations to adult patients should not be extrapolated to the ambulatory care setting as practice models and barriers to vaccine recommendation and administration may be different. Given the morbidity and mortality associated with vaccine-preventable diseases, increasing immunization rates continues to be an important role of the pharmacist.

The goal of this research was to assess the vaccination status of adult patients 19–64 years of age to gather baseline data for designing a pre–post pharmacist intervention study of pharmacist involvement in providing immunization services within three ambulatory care clinics. This internal baseline analysis identifies a potential intervention opportunity for the pharmacist within each of these ambulatory care sites to recommend and administer vaccination during the pharmacy visit.

## Objectives

The objective of this research was to assess the vaccination rates of three ambulatory care pharmacy clinics for patients 19–64 years of age for the following: pneumococcal conjugate (PCV13), pneumococcal polysaccharide (PPSV23), zoster vaccine live (ZVL), hepatitis B (Hep B), and tetanus toxoid, reduced diphtheria toxoid, and acellular pertussis (Tdap) and to compare the vaccination rates for each vaccine between the three clinic sites and to Healthy People 2020 goals.

## Methods

### Design

A cross-sectional study of data collected between January 2016 and March 2017 from three ambulatory care clinic locations (Clinic 1, Clinic 2, and Clinic 3) in a rural community in Virginia evaluated vaccination rates for adult patients 19–64 years of age who attended at least one pharmacy clinic visit at one of the three medical office locations.

Pharmacy clinic appointment calendars were reviewed first to identify patients who were seen in the clinic by the pharmacist. Once a patient was identified as being seen by the pharmacist, the patient’s medical record was then reviewed in reverse chronological order for inclusion criteria. The state IIS was not accessed by the pharmacist and the medical record for each clinic site is not automatically updated with vaccination records from this database. The nursing staff at Clinic 1 routinely evaluates the state IIS at each patient appointment to determine any vaccines the patient may have received by other providers and updates the medical record accordingly. The nursing staff at Clinic 2 and 3 do not routinely evaluate the state IIS, and the pharmacist at each site does not have access to the database. Currently, pharmacists within the clinic sites do not consistently assess patient vaccination status or recommend vaccines that may be needed by patients as a component of pharmacist clinic appointments.

This study was a descriptive analysis of data that provided a simple summary of the vaccination rates of a population sample within three medical practices. The authors hypothesized that vaccination rates for pneumococcal, herpes zoster, Hep B, and Tdap would fall below the national goals set by Healthy People 2020.

The Shenandoah University Institutional Review Board waived written informed consent of subjects and approved this protocol. Each practice site authorized the data collection in advance.

### Participants and setting

Three separate medical practice sites that employ one pharmacy practice faculty member from the Bernard J. Dunn School of Pharmacy at each location were selected to be included in this study. The selections were based on their close geographic location to the school of pharmacy and the access for one-fourth year Advanced Pharmacy Practice Experience (APPE) student to collect the data. Pharmacy faculty members only practiced at their respective sites and patients were only seen in one of the three practice locations. The researchers chose to compare the sites to determine if there were any differences in vaccination rates considering the practices were similar in the services provided by the pharmacist faculty and the sites were within a 2-mile radius of each other. Each practice site includes one pharmacist and up to 10 providers (nurse practitioners, physician assistants, primary care, and general internal medicine physicians) as part of the medical team. Each pharmacist offers patient appointments for ambulatory care services during designated times Monday through Friday for up to 20 patient visits per week. Active promotion or administration of vaccinations by the pharmacist is not a focus of each visit due to practice protocols and provider preference.

Retrospective data collection occurred post-pharmacy clinic visit between January 2016 and March 2017 and specifically during the following time periods for each site: October 2016 to January 2017 (Clinic 1); January 2016 to February 2017 (Clinic 2), and March 2016 to March 2017 (Clinic 3). On average, 15–20 patients are seen at each clinic site per week. The APPE student collected the data during different time periods due to rotation scheduling and the time needed to identify enough patients to be included in the study. This provided a comparable sample size of approximately 80 patients between each clinic site for use in comparison.

Inclusion criteria for the assessment of vaccination status included patients aged 19–64 years who attended at least one pharmacy clinic visit and had at least one chronic condition, as outlined by the ACIP.^16^ Medical conditions considered to be an indication for vaccination included diabetes mellitus, chronic lung disease, chronic cardiovascular disease, liver disease, end-stage renal disease, human immunodeficiency virus (HIV), and other immunocompromising conditions, pregnancy, cigarette smoking, and chronic alcohol use.^16^ Age or chronic condition was identified as an indication for one of the following vaccines: PCV13, PPSV23, ZVL, HepB, and Tdap.

Patients who had at least one pharmacy clinic appointment and met the inclusion criteria based on age or medical condition then had their medical record further reviewed to determine if they had received any of the recommended vaccines. Patient age, gender, medical conditions, chronic cigarette or alcohol use, immunosuppressive medications, and vaccines received were extracted from the medical record and recorded within a Microsoft Excel spreadsheet.^17^ If the recommended vaccine, based on ACIP recommendations, had been received or the patient was contraindicated for the vaccine, the date of receipt or contraindication was recorded within the Excel spreadsheet. An alphanumeric number was assigned to each patient within the medical record to ensure there were no duplicates in data collection and no patient identifiers were captured. This assigned number was recorded within the Excel spreadsheet which served as the official record of data collection.

To maintain consistency among clinic samples, provider preferences, practice protocols or standing orders, the US Food and Drug Administration (FDA) approved indication and vaccine product labeling were not considered as an indication for vaccination. In addition, data were collected for patients who received a vaccine in the absence of an ACIP indication but were excluded from the results.

### Statistical analysis

Vaccination status was determined based on the 2017 ACIP recommendations which were the current recommendations at the time of this review.^16^ Differences in vaccination rates for each vaccine between each clinic were evaluated using Pearson’s *x*^2^-squared analysis in SPSS statistical software version 25 (IBM).^18^ A *p*-value of < 0.05 indicated statistical significance. Since this study was a retrospective cross-sectional evaluation of data without intent to assess superiority or inferiority, sample size calculations were not performed. Rather, the sample size was a result of eligible patients based on inclusion criteria at the three sites assessed.

## Results

A total number of 499 patients from all clinic sites were screened for inclusion in the study. Of the total number of patients screened, there were 240 patients between 19 and 64 years of age with a mean age of 52.8 (± 9.15) years (Table 1). Patient gender was equally represented with 53% (*n* = 128) male and 47% (*n* = 112) female. Patients who were excluded from the assessment included 258 patients who were over 64 years of age and one patient who was less than 19 years of age. The majority of patients, 90% (*n* = 216), in all three clinics were referred to the pharmacist for management of diabetes or pre-diabetes.

**Table 1.** Demographics and health conditions of patients included, *n* (%).

	Clinic 1	Clinic 2	Clinic 3	Total
Age				
19–64 (years)	80	79	81	240
Years, mean (SD)	53 (9)	53 (9.3)	52.5 (9.15)	52.8 (9.15)
Range (years)	28–64	24–64	28–64	24–64
Gender, *n* (%)				
Male	47 (58.7)	46 (58.2)	35 (43.2)	128 (53.3)
Female	33 (41.3)	33 (41.7)	46 (56.7)	112 (46.6)
Health condition, *n* (%)				
Diabetes mellitus	64 (80)	71 (89.9)	81 (100)	216 (90)
Chronic lung disease	10 (12.5)	13 (16.5)	9 (11.1)	32 (13.3)
Chronic CV disease	5 (6.3)	3 (3.8)	3 (3.7)	11 (4.6)
Liver disease	2 (2.5)	19 (24)	2 (2.5)	23 (9.6)
ESRD	0	0	0	0
HIV	0	0	1 (1.2)	1 (0.4)
Other immunocompromising conditions^a^	3 (3.8)	3 (3.8)	6 (7.4)	12 (5)
Pregnancy	0	0	1 (1.2)	1 (0.4)
Cigarette smoker	12 (15)	15 (19)	25 (30.9)	52 (21.7)
Chronic alcohol use	5 (5)	3 (3.8)	1 (1.2)	8 (3.3)

SD: standard deviation; CV: cardiovascular; ESRD: end stage renal disease; HIV: human immunodeficiency virus.

Total number of patients screened for inclusion at each site: Clinic 1 (*n* = 176); Clinic 2 (*n* = 161); and Clinic 3 (*n* = 162).

a As defined by ACIP and recommended immunization schedule for adults aged 19 years or above.^12^

Vaccination rates for each vaccine for each clinic and the combined vaccination rate for each vaccine are shown in Table 2. There was a statistically significant difference in the rate of vaccination between all three clinic sites for PCV13 (*p* = 0.002), PPSV23 (*p* < 0.001), HepB (*p* < 0.001), and Tdap (*p* < 0.001). No statistically significant difference was noted in the rate of vaccination for ZVL between all clinic sites (*p* = 0.12).

**Table 2.** Clinic and total vaccination rates per vaccine, n^a^ (%).

Vaccine	Clinic 1	Clinic 2	Clinic 3	Total	HP 2020 Goal^b^	*p-*value^c^
Pneumococcal conjugate (PCV 13)	3/3 (100)	0/3 (0)	0/6 (0)	3/12 (25)	(90)	0.002
Pneumococcal polysaccharide (PPSV23)	23/68 (33.8)	43/73 (58.9)	13/80 (16.3)	79/221 (35.7)	(90)	<0.001
Zoster vaccine live (ZVL)	5/22 (22.7)	4/24 (16)	9/21 (42.9)	18/67 (26.9)	(30)	0.12
Hepatitis B (HepB)	10/49 (20.4)	0/49 (0)	0/59 (0)	10/157 (6.4)	N/A	<0.001
Tetanus toxoid, reduced diphtheria toxoid, and acellular pertussis (Tdap)	32/80 (40)	56/78 (71.8)	33/81 (40.7)	121/239 (50.6)	N/A	<0.001

HP: Healthy People; N/A: not applicable; ACIP: Advisory Committee on Immunization Practices.

a Expressed as number of patients vaccinated per total number of patients with an ACIP indication for the vaccine.

b Goals reported as a percentage and outlined by Healthy People 2020.^4^

c Pearson’s Chi-square was used to determine significance, defined as *p* < 0.05, between Clinics 1, 2, and 3.

The overall combined vaccination rates of patients included for the three clinic sites were below established Healthy People 2020 goals for pneumococcal and ZVL as presented in Table 2. Specific goals for Tdap and HepB are not outlined by Healthy People 2020 for this target population; therefore, a comparison of combined vaccination rates for the clinic sites was not evaluated.

Patients who received a vaccine based on product labeling or FDA indication, but not according to ACIP recommendations, were recorded but excluded from the analysis. However, 4 patients had received ZVL who were 50–59 years of age and 17 patients were vaccinated with pneumococcal conjugate (PCV13) vaccine in absence of an ACIP recommendation by other medical providers within the practice.

## Discussion

Despite ACIP recommendations that all health care providers review vaccination history as a standard of practice, the vaccination rates of each clinic site are below established goals. Furthermore, pharmacists within the clinic sites outlined in this study are not actively engaged in assessing the vaccination status of patients during clinic appointments as this is not within their established protocols for the pharmacy clinic visit. This may contribute to why vaccination rates are below goal and highlights an opportunity for the pharmacist to be more involved in the immunization program within each practice site. The pharmacist working within the medical office space is positioned to assess vaccination status and educate patients on the importance of vaccination. Expanding the role of these pharmacists to include recommendation and administration of vaccination at that clinical encounter may lead to increased vaccination rates.

The significant differences in vaccination rates between clinics may be due to several reasons, however, were not specifically studied. Providers within each site may share the same opinions regarding barriers that other clinicians have reported in the published literature. Perhaps these providers also believe that assessing and administering vaccinations to adult patients is outside their scope of practice or they do not have the time and rely on other providers to perform this function. There could be a lack of appropriate storage to maintain an adequate inventory for all vaccines. Or, it is possible that the providers lack the expertise with current recommendations, which prevents them from assessing the vaccination status of their patients. In addition, individual provider recommendations may differ from ACIP recommendations since they have the autonomy to make clinical decisions that may fall outside of accepted guidelines. This may also explain why patients received a vaccine despite an ACIP indication. Patient refusal, vaccine reimbursement, and patient cost may contribute to the lack of provider recommendations for vaccination within these medical practices.

The majority of patients included in the study were referred to the pharmacist clinic for the management of diabetes which carries an indication for PPSV23 and HepB and these pharmacists were not actively engaged in the recommendation or administration of vaccines. The vaccination rate for PPSV23 for each clinic was below Healthy People 2020 goals and there was a statistically significant difference in the vaccination rate for PPSV23 and HepB between sites. This presents an opportunity for the pharmacist at each site to recommend PPSV23 and HepB during the clinic visit. While it is possible that these patients have been offered and refused these vaccinations previously, this study did not assess this. It is difficult to determine why the rates for PPSV23 are so different between the sites; therefore, assumptions based on published literature that highlights barriers to increasing vaccination rates may be considered.

Very few PCV13 vaccines were administered to eligible patients, which could be due to the timeframe of when the new recommendations for this vaccine were released by ACIP as well as a lack of expertise in providing PCV13 to adult patients. Traditionally, PPSV23 was the only vaccine recommended for this patient group. Lack of understanding regarding the new recommendations or vaccine reimbursement may have contributed to patients not receiving PCV13.

When comparing the rate of vaccination for other vaccines within these sites, HepB seems to be recommended less, considering the number of patients who are eligible and the zero-percentage rate for two clinics. This presents another opportunity for the pharmacist to recommend HepB during clinic visits to capture those who have never been recommended or refused to receive the vaccine.

Vaccination rates for ZVL were not statistically different between sites. Insurance coverage for ZVL under Medicare Part D and commercial insurance plans is well supported by pharmacists vaccinating adult patients against herpes zoster within community pharmacies and may be one reason these rates were comparable. In addition, the improved marketing of this vaccine by the manufacturer may have also contributed to the comparable rates between each site. Zoster Vaccine Recombinant (RZV) was not yet approved by the FDA at the time of the study and therefore not included in the study data and results. The vaccination rate for Tdap was 40% or higher for each clinic but statistically different between sites. It appears that providers within each site are recommending and administering this vaccine with good outcomes and the statistically significant difference between sites may only be due to a patient’s willingness to receive Tdap.

Based on the information obtained in this study, it is difficult to determine the specific reasons why the rates for each vaccine are so different between the sites. Further exploration of provider opinions regarding vaccination services within each site as well as documentation of patients’ refusal to vaccinations may identify practice-specific reasons for low vaccination rates. With the implementation of a pharmacist at these medical offices, additional studies are recommended to determine if the pharmacists can help increase vaccination rates and decrease barriers that clinicians have described when protocols include the administration of vaccines. The collaboration of pharmacists within the medical office space at each clinic site may enhance the current vaccination programs.

The authors recognize the findings of the three practice sites may not be representative of other ambulatory care settings in which a pharmacist provides patient care services. Furthermore, our results may not reference rates of vaccination from other providers (e.g. previous health care provider, community pharmacy, or health department) as reporting of vaccination status for adult patients is not consistently updated in one database. The state IIS was not checked by the pharmacist during data collection, which potentially missed additional vaccination history within the patients’ medical record that may not have been up-to-date prior to the data collection. The majority of patients evaluated had diabetes (90%), which is representative of the types of patients referred to the pharmacists’ service but may be considered sampling bias. Although this highlights that the pharmacist within each practice setting may contribute to the low rate of vaccination within this patient population, since they are not currently making appropriate recommendations during their visits. Reviewing all patients within each practice site who met the inclusion criteria, despite an appointment with the pharmacist, would give an overall picture of vaccination rates of patients within the medical practice sites.

Additional limitations have been identified relating to the design and methods used. Calendar appointments were not reviewed during the same time period. As such, the vaccination status of patients may have changed during the overall data collection period of the study if they received a vaccine from a different provider and the information was updated in the patient’s medical record. The assessment of patients over a defined period of time (e.g. at least one appointment within the past 3 months) for all clinic sites may have minimized potential sources of variation. In addition, the results did not account for patient refusal or financial difficulties that may have prevented vaccination despite a previous provider recommendation.

Regardless of the limitations, variations in vaccination rates in patients 19–64 years of age within three separate ambulatory care pharmacy clinic sites were identified, despite clear recommendations outlined by ACIP. The findings from this study provide additional awareness regarding the disparity in vaccine recommendations for adult patients aged 19–64 years and the significant need for pharmacists to provide these services. Hopefully, the data presented within this study will validate the need for continued discussion regarding pharmacists’ involvement in comprehensive vaccination programs.

## Conclusion

Patients 19–64 years of age remain at risk for vaccine-preventable diseases, despite ACIP recommendations, practice protocols, coverage under the ACA, and increased access points for vaccination services. While barriers continue to exist with vaccination recommendations and administration, adult patients remain unprotected against vaccine-preventable diseases. One may claim that vaccination screening and administration is a standard of care for all practitioners regardless of practice setting. However, the literature continues to report low vaccination rates and there is a lack of documented pharmacist interventions and outcomes data.

This baseline data reporting low vaccination rates in three ambulatory care pharmacy clinics should serve as a call to action to all health care providers in the ambulatory care setting and presents an opportunity for ambulatory care pharmacists to become more involved in their practice site’s vaccination program or to document current activities. Outcome evaluations of pharmacist interventions in recommending and administering vaccinations during ambulatory care clinic appointments are needed to determine their impact on vaccination rates.
